# Supplementary material for: The anti-biofilm effect of silver-nanoparticle-decorated quercetin nanoparticles on a multi-drug resistant Escherichia coli strain isolated from a dairy cow with mastitis
Source: PeerJ. 2018 Oct 16;6:e5711. doi: 10.7717/peerj.5711 (PMC6195112; doi:10.7717/peerj.5711)
Supplement: Supplemental Information 1 [file peerj-06-5711-s001.zip › Supplemental Files/File 1/The survival rate-raw data.docx]

**Table 1-1** **The survival raw data of *E. coli* ECDCM1 with different concentration QA NPs**

| The different concentration QA NPs | The dilution folds | The raw data of *E. coli* ECDCM1 CFU | | |
| --- | --- | --- | --- | --- |
| 0µg/ml | 10^-6^ | 116 | 107 | 111 |
| 0.5µg/ml | 10^-6^ | 99 | 107 | 103 |
| 0µg/ml | 10^-6^ | 116 | 107 | 111 |
| 1.0µg/ml | 10^-6^ | 61 | 65 | 58 |
| 0µg/ml | 10^-6^ | 94 | 85 | 78 |
| 5.0µg/ml | 10^-4^ | 1050 | 1110 | 1015 |
| 0µg/ml | 10^-6^ | 114 | 125 | 112 |
| 10.0µg/ml | 10^-1^ | 0 | 0 | 0 |

**Table 1-2** **The survival raw data of *E. coli* ECDCM1 with different concentration QA NPs**

| The different concentration QA NPs | The dilution folds | The raw data of *E. coli* ECDCM1 CFU | | |
| --- | --- | --- | --- | --- |
| 0µg/ml | 10^-6^ | 106 | 99 | 81 |
| 0.5µg/ml | 10^-6^ | 99 | 89 | 84 |
| 0µg/ml | 10^-6^ | 114 | 125 | 112 |
| 1.0µg/ml | 10^-6^ | 73 | 65 | 66 |
| 0µg/ml | 10^-6^ | 140 | 155 | 157 |
| 5.0µg/ml | 10^-5^ | 271 | 267 | 255 |
| 0µg/ml | 10^-6^ | 106 | 99 | 81 |
| 10.0µg/ml | 10^0^ | 0 | 0 | 0 |

**Table 1-3** **The survival raw data of *E. coli* ECDCM1 with different concentration QA NPs**

| The different concentration QA NPs | The dilution folds | The raw data of *E. coli* ECDCM1 CFU | | |
| --- | --- | --- | --- | --- |
| 0µg/ml | 10^-6^ | 140 | 155 | 157 |
| 0.5µg/ml | 10^-6^ | 151 | 134 | 136 |
| 0µg/ml | 10^-6^ | 123 | 130 | 136 |
| 1.0µg/ml | 10^-6^ | 72 | 69 | 66 |
| 0µg/ml | 10^-6^ | 85 | 98 | 96 |
| 5.0µg/ml | 10^-4^ | 1172 | 1430 | 1274 |
| 0µg/ml | 10^-6^ | 153 | 169 | 187 |
| 10.0µg/ml | 10^-1^ | 0 | 0 | 0 |

**Table 2-1 The survival raw data of *E. coli* ECDCM1 with different concentration Ag NPs**

| The different concentration Ag NPs | The dilution folds | The raw data of *E. coli* ECDCM1 CFU | | |
| --- | --- | --- | --- | --- |
| 0µg/ml | 10^-6^ | 158 | 142 | 166 |
| 0.5µg/ml | 10^-6^ | 153 | 155 | 163 |
| 0µg/ml | 10^-6^ | 140 | 155 | 157 |
| 1.0µg/ml | 10^-6^ | 139 | 149 | 156 |
| 0µg/ml | 10^-6^ | 123 | 130 | 136 |
| 5.0µg/ml | 10^-6^ | 133 | 145 | 133 |
| 0µg/ml | 10^-6^ | 114 | 125 | 112 |
| 10.0µg/ml | 10^-5^ | 623 | 595 | 593 |

**Table 2-2 The survival raw data of *E. coli* ECDCM1 with different concentration Ag NPs**

| The different concentration Ag NPs | The dilution folds | The raw data of *E. coli* ECDCM1 CFU | | |
| --- | --- | --- | --- | --- |
| 0µg/ml | 10^-6^ | 111 | 136 | 134 |
| 0.5µg/ml | 10^-6^ | 119 | 135 | 116 |
| 0µg/ml | 10^-6^ | 128 | 138 | 142 |
| 1.0µg/ml | 10^-6^ | 123 | 123 | 140 |
| 0µg/ml | 10^-6^ | 158 | 142 | 166 |
| 5.0µg/ml | 10^-6^ | 177 | 149 | 156 |
| 0µg/ml | 10^-6^ | 99 | 108 | 106 |
| 10.0µg/ml | 10^-5^ | 543 | 577 | 547 |

**Table 2-3 The survival raw data of *E. coli* ECDCM1 with different concentration Ag NPs**

| The different concentration Ag NPs | The dilution folds | The raw data of *E. coli* ECDCM1 CFU | | |
| --- | --- | --- | --- | --- |
| 0µg/ml | 10^-6^ | 112 | 103 | 104 |
| 0.5µg/ml | 10^-6^ | 124 | 112 | 108 |
| 0µg/ml | 10^-6^ | 158 | 142 | 166 |
| 1.0µg/ml | 10^-6^ | 137 | 179 | 135 |
| 0µg/ml | 10^-6^ | 195 | 183 | 160 |
| 5.0µg/ml | 10^-6^ | 173 | 191 | 193 |
| 0µg/ml | 10^-6^ | 128 | 138 | 142 |
| 10.0µg/ml | 10^-5^ | 760 | 661 | 695 |

**Table 3-1 The survival raw data of *E. coli* ECDCM1 with different concentration Qe**

| The different concentration Qe | The dilution folds | The raw data of *E. coli* ECDCM1 CFU | | |
| --- | --- | --- | --- | --- |
| 0µg/ml | 10^-6^ | 116 | 107 | 111 |
| 0.5µg/ml | 10^-6^ | 110 | 138 | 123 |
| 0µg/ml | 10^-6^ | 116 | 107 | 111 |
| 1.0µg/ml | 10^-6^ | 125 | 125 | 118 |
| 0µg/ml | 10^-6^ | 94 | 85 | 78 |
| 5.0µg/ml | 10^-6^ | 101 | 104 | 85 |
| 0µg/ml | 10^-6^ | 114 | 125 | 112 |
| 10.0µg/ml | 10^-6^ | 97 | 77 | 86 |

**Table 3-2 The survival raw data of *E. coli* ECDCM1 with different concentration Qe**

| The different concentration Qe | The dilution folds | The raw data of *E. coli* ECDCM1 CFU | | |
| --- | --- | --- | --- | --- |
| 0µg/ml | 10^-6^ | 156 | 140 | 139 |
| 0.5µg/ml | 10^-6^ | 184 | 139 | 128 |
| 0µg/ml | 10^-6^ | 114 | 109 | 105 |
| 1.0µg/ml | 10^-6^ | 130 | 130 | 123 |
| 0µg/ml | 10^-6^ | 123 | 130 | 136 |
| 5.0µg/ml | 10^-6^ | 146 | 142 | 141 |
| 0µg/ml | 10^-6^ | 153 | 169 | 187 |
| 10.0µg/ml | 10^-6^ | 126 | 122 | 137 |

**Table 3-3 The survival raw data of *E. coli* ECDCM1 with different concentration Qe**

| The different concentration Qe | The dilution folds | The raw data of *E. coli* ECDCM1 CFU | | |
| --- | --- | --- | --- | --- |
| 0µg/ml | 10^-6^ | 100 | 96 | 90 |
| 0.5µg/ml | 10^-6^ | 120 | 112 | 107 |
| 0µg/ml | 10^-6^ | 99 | 127 | 118 |
| 1.0µg/ml | 10^-6^ | 106 | 147 | 126 |
| 0µg/ml | 10^-6^ | 110 | 134 | 139 |
| 5.0µg/ml | 10^-6^ | 154 | 153 | 135 |
| 0µg/ml | 10^-6^ | 150 | 170 | 172 |
| 10.0µg/ml | 10^-6^ | 114 | 123 | 128 |
