# Supplementary figures and images for: The anti-biofilm effect of silver-nanoparticle-decorated quercetin nanoparticles on a multi-drug resistant Escherichia coli strain isolated from a dairy cow with mastitis
Source: PeerJ. 2018 Oct 16;6:e5711. doi: 10.7717/peerj.5711 (PMC6195112; doi:10.7717/peerj.5711)

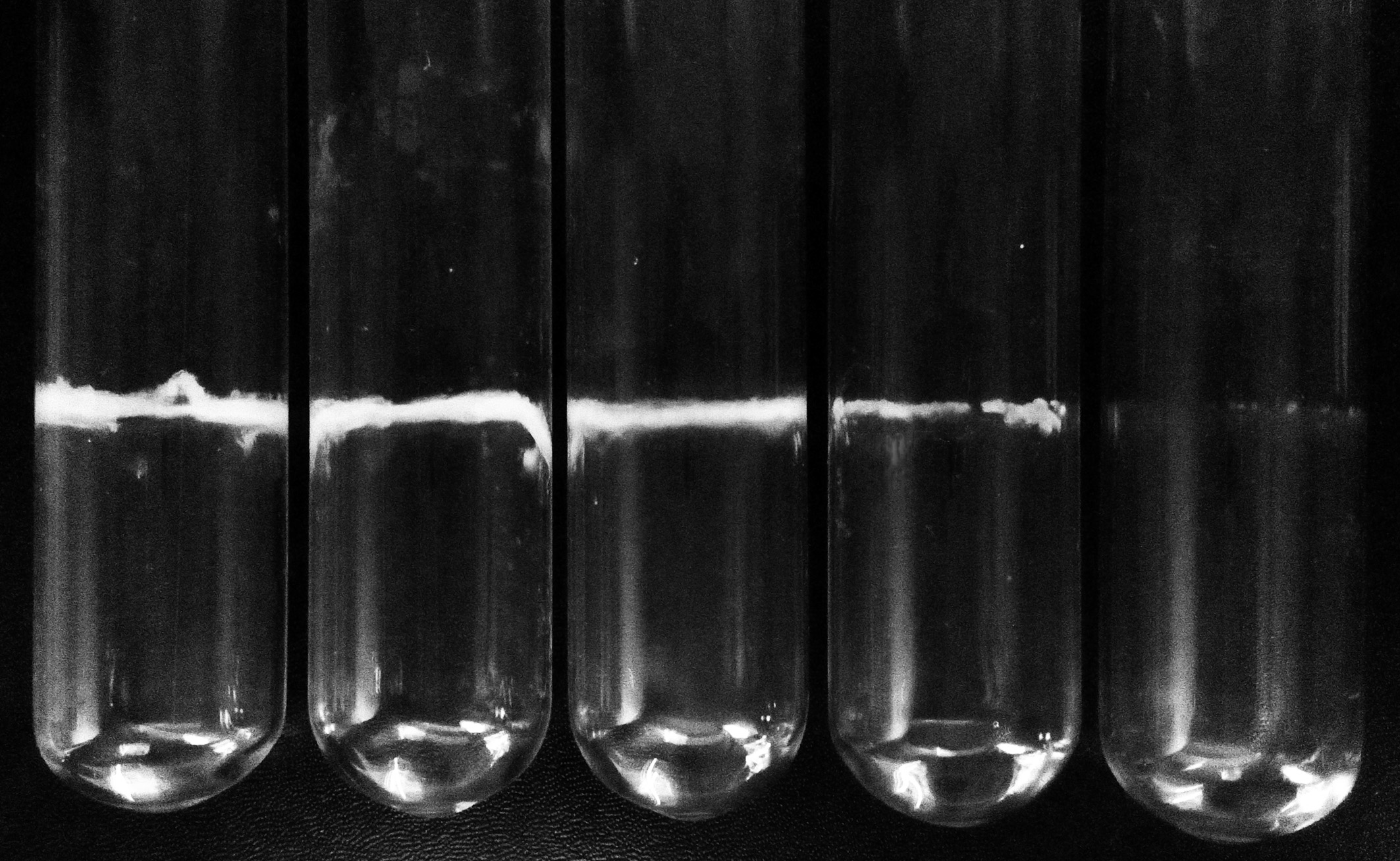

Supplement: Supplemental Information 1 [file peerj-06-5711-s001.zip › Supplemental Files/File 2/Biofilm-AgNPs.jpg]

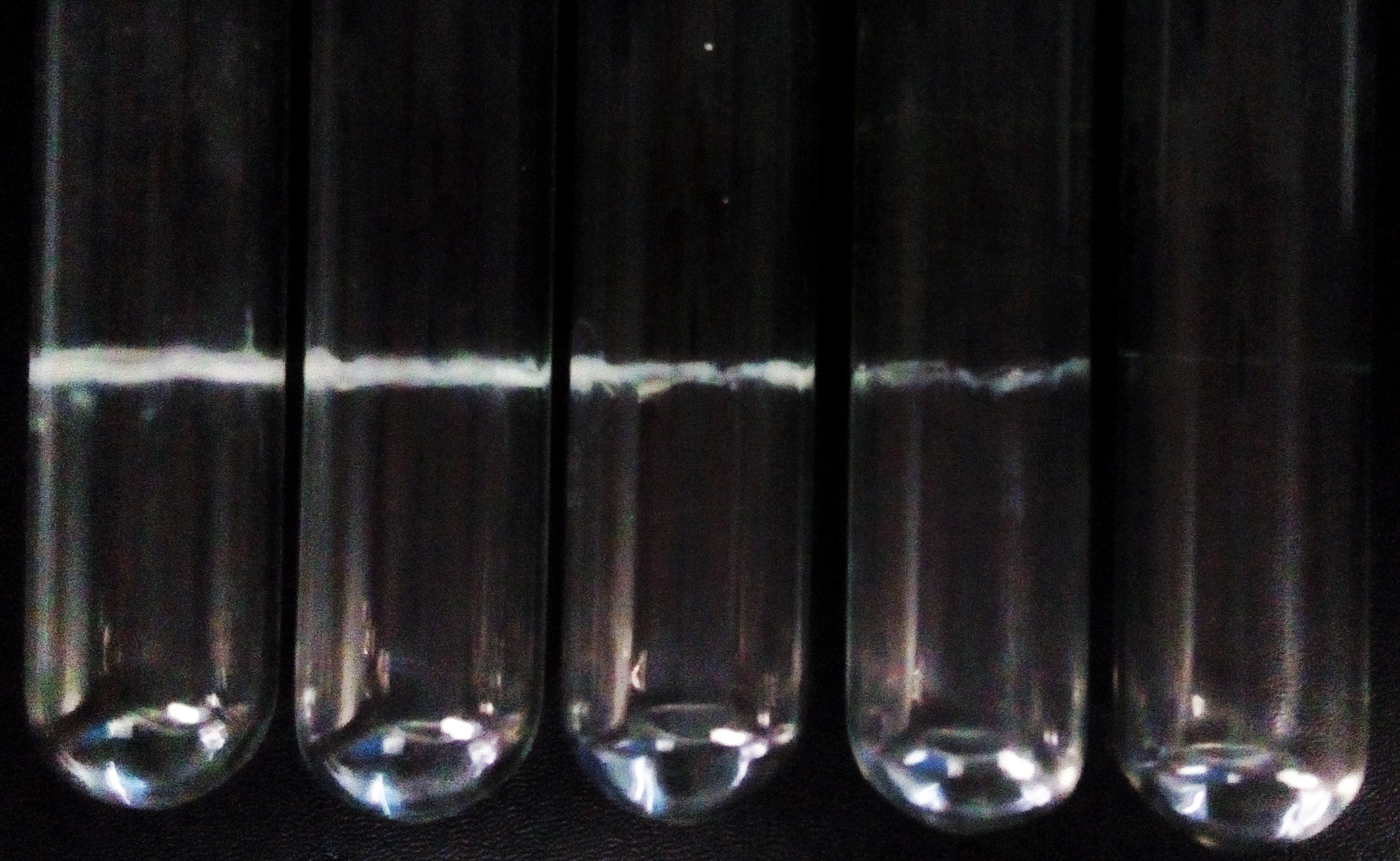

Supplement: Supplemental Information 1 [file peerj-06-5711-s001.zip › Supplemental Files/File 2/Biofilm-QA.jpg]

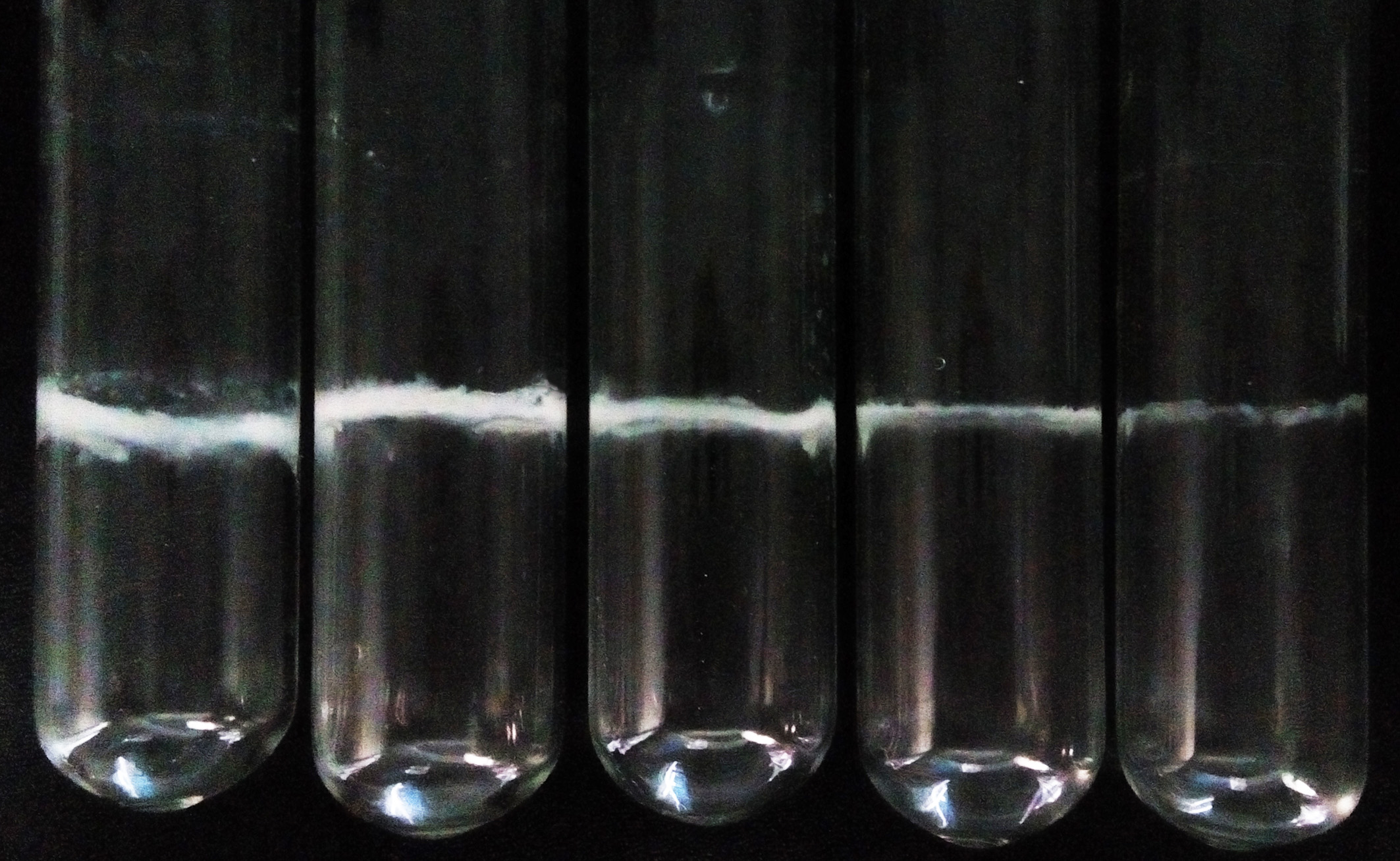

Supplement: Supplemental Information 1 [file peerj-06-5711-s001.zip › Supplemental Files/File 2/Biofilm-Qe.jpg]

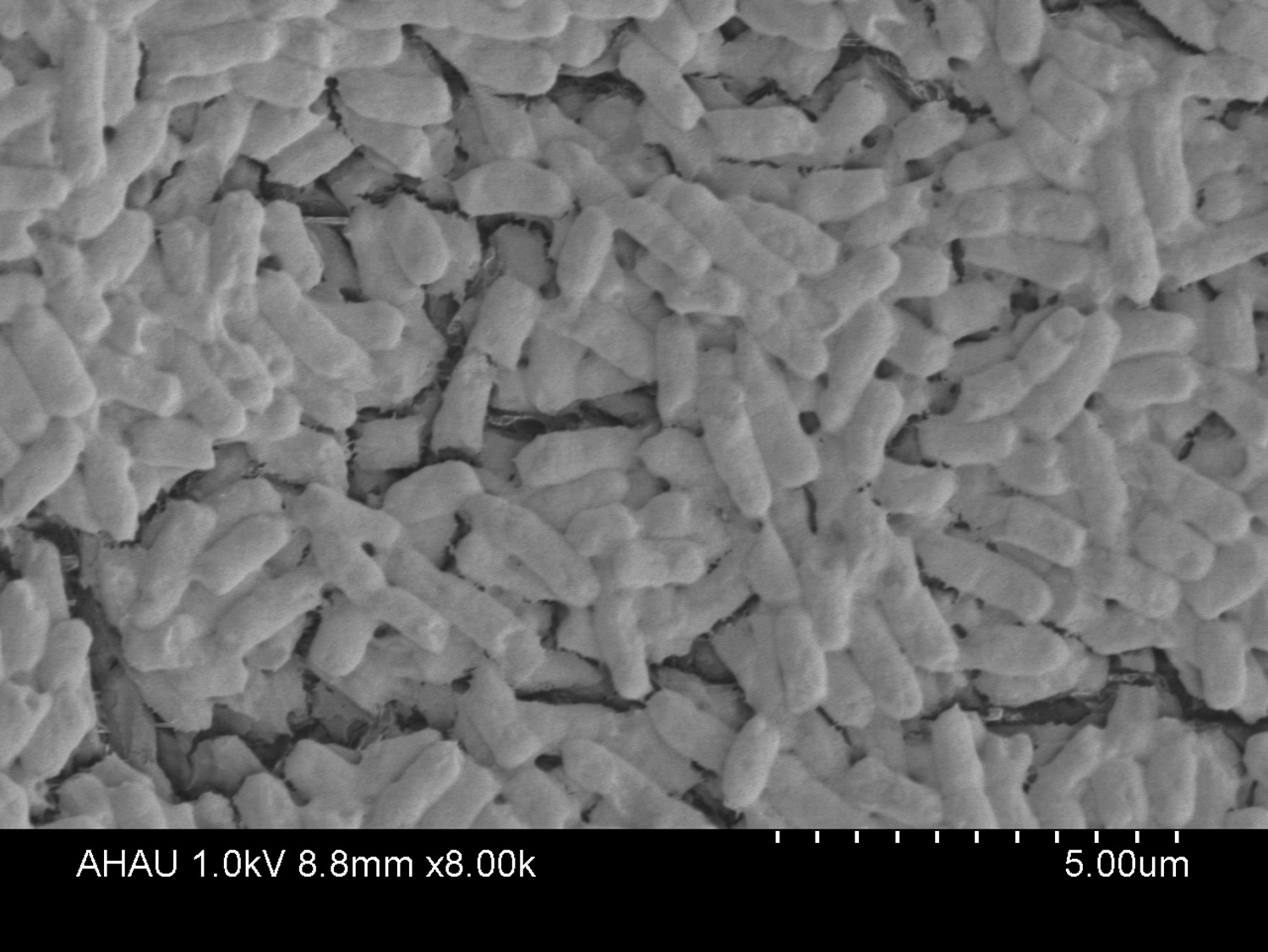

Supplement: Supplemental Information 1 [file peerj-06-5711-s001.zip › Supplemental Files/File 4/SEM-AgNPs.jpg]

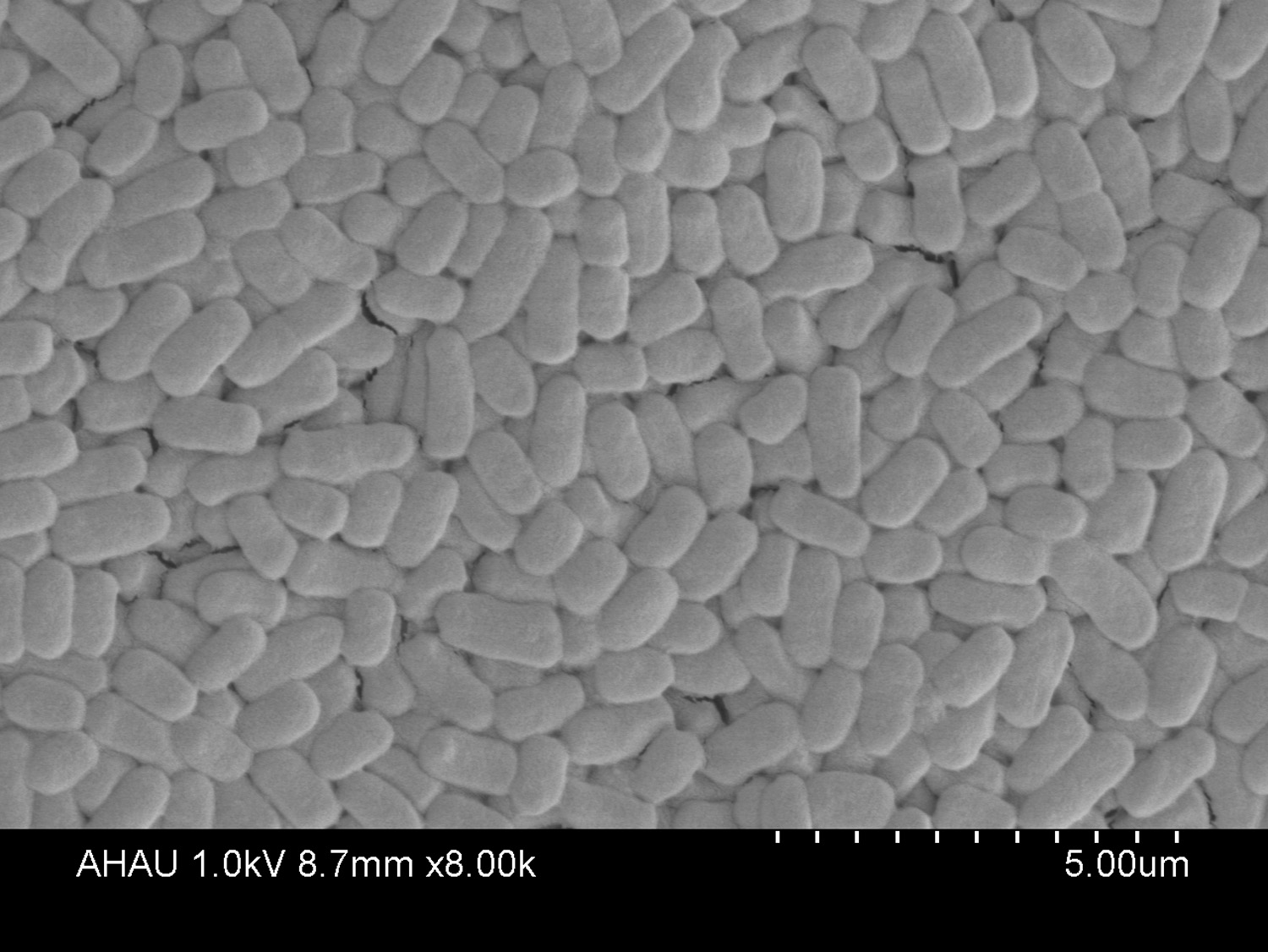

Supplement: Supplemental Information 1 [file peerj-06-5711-s001.zip › Supplemental Files/File 4/SEM-NTC.jpg]

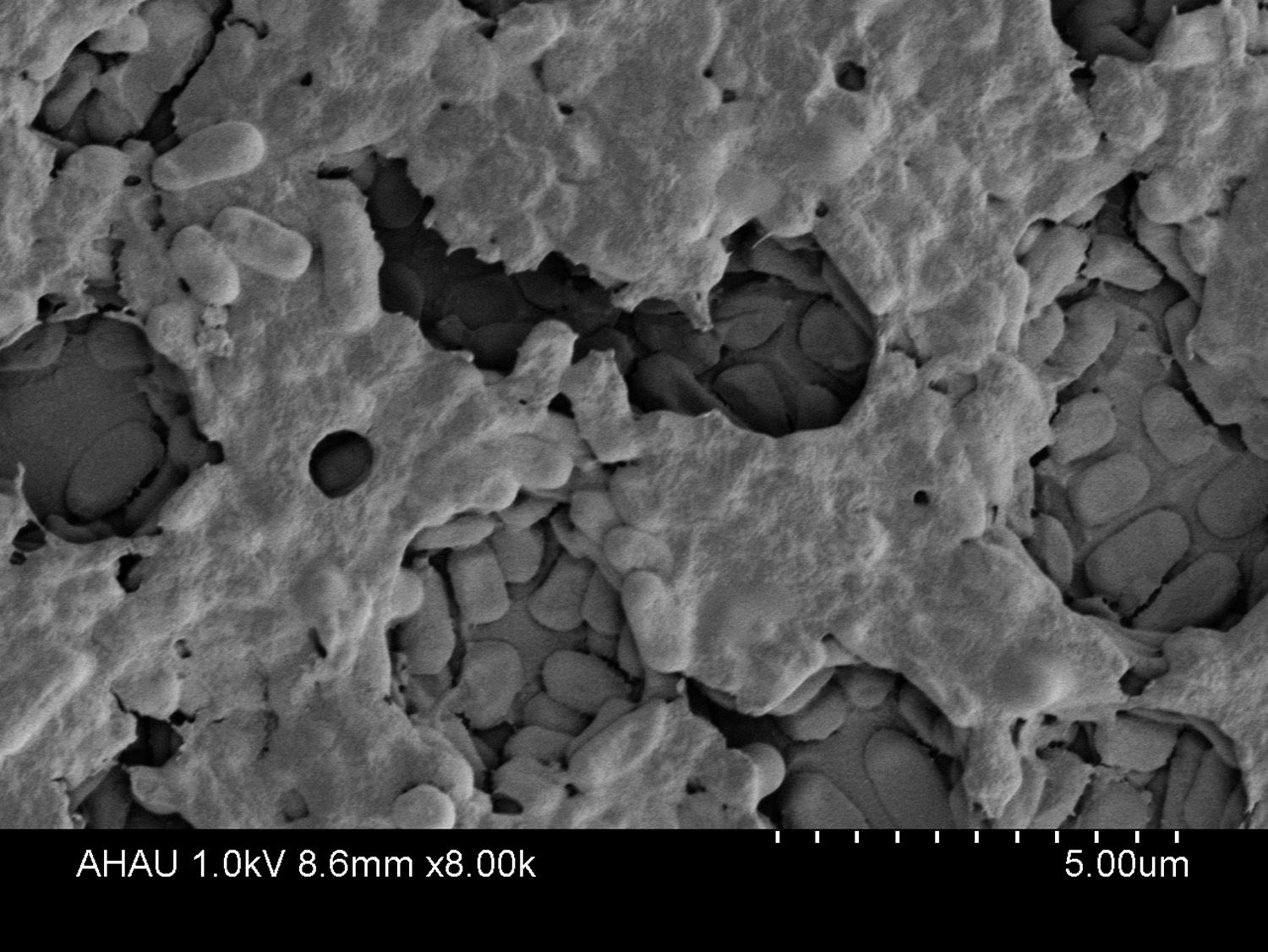

Supplement: Supplemental Information 1 [file peerj-06-5711-s001.zip › Supplemental Files/File 4/SEM-QANPs.jpg]

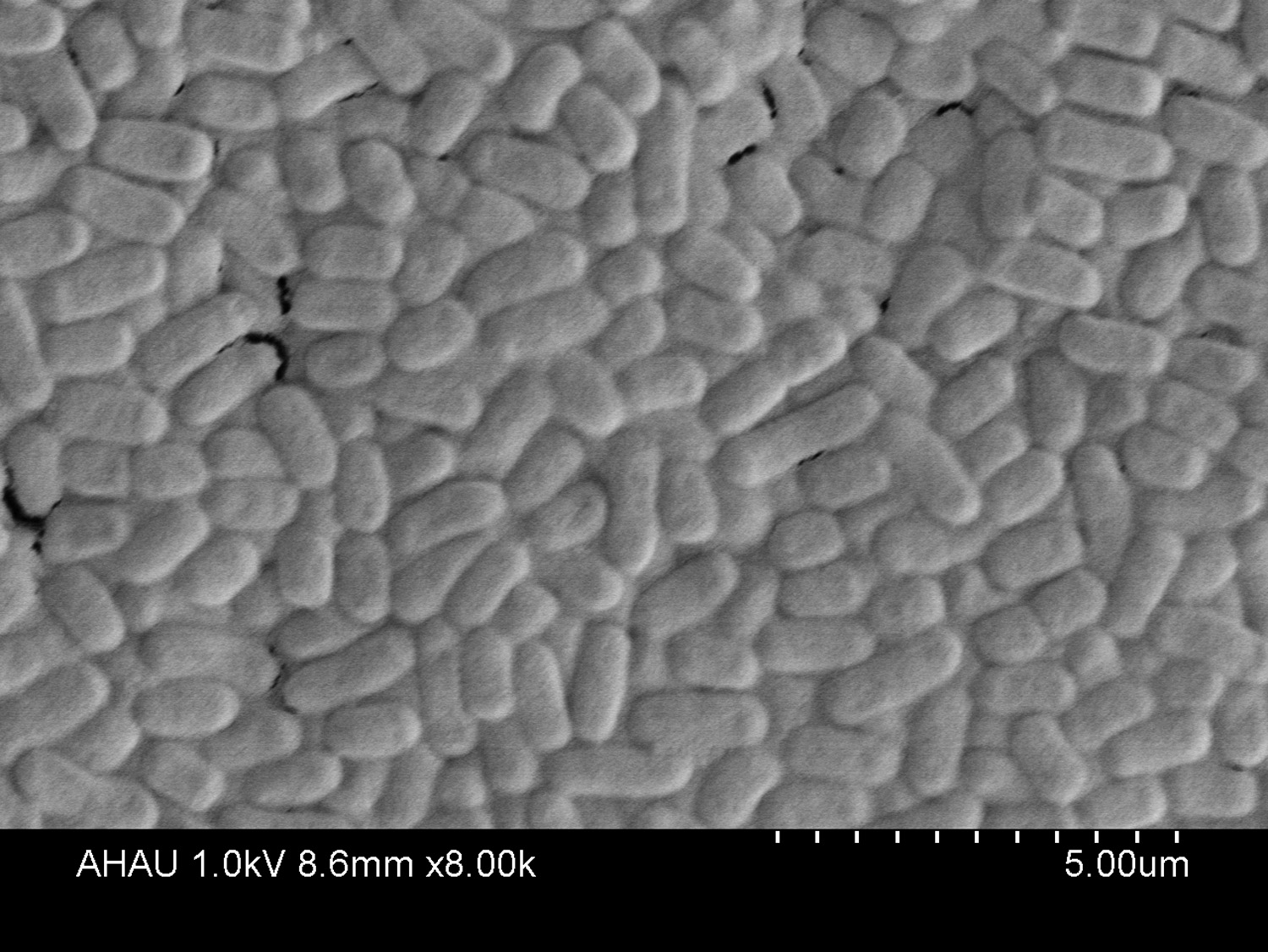

Supplement: Supplemental Information 1 [file peerj-06-5711-s001.zip › Supplemental Files/File 4/SEM-Qe.jpg]
